# Post–Synthetic Modification of MOF–808 for Mixed Matrix Membranes with High and Stable Ion Separation Capacity

**DOI:** 10.3390/molecules30234554

**Published:** 2025-11-26

**Authors:** Bahar Karadeniz, Han-Liang Fang, Yi-Ying He, Qi-Lin Ye, Jun-Yu Chen, Jian Lü

**Affiliations:** 1 Fujian Provincial Key Laboratory of Soil Environmental Health and Regulation, College of Resources and Environment, Fujian Agriculture and Forestry University, Fuzhou 350002, China; bahar.karadeniz@irb.hr (B.K.); fanghanliang2023@163.com (H.-L.F.); heyiying00@163.com (Y.-Y.H.); 17345151886@163.com (Q.-L.Y.); 2 Ruđer Bošković Institute, Bijenička Cesta 54, 10000 Zagreb, Croatia; 3 FAFU–DAL Joint College (International College), Fujian Agriculture and Forestry University, Fuzhou 350108, China

**Keywords:** metal–organic framework, mixed matrix membrane, ion separation, water flux

## Abstract

The global clean water crisis is a pressing sustainable development challenge that demands urgent solutions. Membrane separation technology has emerged as a leading approach for seawater desalination, offering great potential to address freshwater scarcity. However, achieving both high water flux and high salt rejection in desalination membranes remains a major challenge. Mixed matrix membranes (MMMs), which combine polymer substrates with functional fillers, have shown promise, but their performance is often limited by poor compatibility between the embedded materials and the polymer matrix. In this work, a post-synthetic modification of the metal–organic framework MOF–808 was carried out to improve the interfacial compatibility between the modified MOF–808–SP and polyethersulfone substrate. Remarkably, increasing the loading of MOF–808–SP sustained the membrane selectivity while simultaneously enhancing water flux. This performance contrasts with membranes containing unmodified MOF–808, highlighting the crucial role of improved MOF–polymer compatibility in achieving synergistic separation performance.

## 1. Introduction

Considering the ever-growing scarcity of water resources, the demand for clean water is further aggravated by the overwhelming proportion of global reserves existing as largely untapped seawater [1]. Consequently, the profound significance lies in the conversion of seawater into portable freshwater. This has thus far been achieved primarily through energy-intensive desalination strategies. Although these conventional methods yield appreciable quantities of clean water, substantial energy expenditure and environmental contamination thereby arise. Therefore, seeking more efficient and eco-friendly approaches to seawater treatment is of great importance [2].

Among the various technologies for seawater desalination, membrane-based treatment [3] is particularly promising due to its effective separation of pollutants and undesirable components from seawater, combined with its relatively low processing cost. Furthermore, membranes can be readily optimized, offering an efficient route to convert seawater into freshwater without additional processing steps or chemical reagents. In the desalination industry, commercially available reverse osmosis (RO) membranes are predominantly thin film composite (TFC) membranes [4] consisting of a selective separation layer supported by a porous substrate. Typically, the selective layer is a polyamide (PA) film formed via the interfacial polymerization (IP) reaction [5,6]. However, like most membrane materials, PA membranes inherently suffer from a permeability selectivity trade-off, where an increase in water permeability is generally accompanied by an undesirable decline in salt rejection.

To overcome the fundamental trade-off between membrane permeability and selectivity, numerous strategies have been explored. Among these, mixed matrix membranes [7,8,9] (MMMs) have emerged as a promising solution, as they combine the advantages of the polymeric substrate and the filler material. A practical approach involves incorporating various nanomaterials with diverse physicochemical properties into the selective polyamide layer, thus enhancing the structural and functional performance of MMMs. The judicious selection of nanomaterials in MMMs is therefore crucial [10,11,12]. Dong et al. [13] embedded NaY zeolite nanoparticles into the PA layer via interfacial polymerization to form a novel zeolite–polyamide thin film nanocomposite (TFN) membrane. Under optimized conditions, the TFN membranes demonstrated a significantly improved separation performance for brackish water desalination, with nearly double the water flux and high salt rejection. However, the membrane exhibited an irreversible decrease in salt rejection with an increasing dosage of NaY nanoparticles. Similarly, Liu et al. [14] incorporated MCM–48 nanoparticles into PA membranes, which enhanced water flux proportionally with increasing nanoparticle content, but at the expense of salt rejection, which decreased from 95% to 80%. The reduction in salt rejection observed in such cases can be attributed to nanogaps or interfacial defects between the nanoparticles and the membrane substrate [15]. Therefore, enhancing the compatibility between the dopant material and the membrane substrate is crucial for optimizing the performance of MMMs.

Metal–organic frameworks (MOFs) [16] have attracted considerable research attention due to their unique properties, including tunable pore volume and surface characteristics. Their intrinsic pore structures can serve as low-resistance channels that facilitate the rapid transport of water molecules, while the dense PA layer provides selective molecular sieving [17]. This synergistic effect makes MOF-based MMMs promising candidates for ion separation. For example, MMMs incorporating MOFs such as MIL–101 (Cr) [18,19], ZIF–8 [20], and UiO–66–NH_2_ [21] have demonstrated excellent performance owing to their high surface area and suitable pore sizes. In the case of MIL–101 (Cr), embedding the MOF within the membrane substrate introduces additional water transport pathways. At a loading of 0.05% *w*/*v* MIL–101 (Cr), water permeability increased by 44% while maintaining nearly unchanged salt rejection. However, excessive MIL–101 (Cr) loading resulted in a marked decrease in salt rejection, likely due to interfacial defects forming between the PA layer and the MOF [22,23]. To address this, tailoring the surface properties of MOFs has been shown to minimize such interfacial defects [24], enabling the simultaneous realization of high-water flux and salt rejection in MOF-based MMMs. Therefore, enhancing the compatibility between the dopant material and the membrane substrate is crucial for optimizing the performance of MMMs. Despite extensive research on MOF-based MMMs, studies involving MOF–808 remain limited, particularly in the context of tailored interfacial chemistry [25,26,27]. Many reported approaches employ pristine MOFs without specific surface functionalization, which often leads to poor dispersion within polymer matrices and interfacial defects that hinder ion selectivity [28,29]. To address this gap, our work introduces a novel post-synthetic modification of MOF–808 using spiropyran (SP) molecules bearing terminal carboxyl groups. Unlike previous MOF–808 functionalizations that primarily target gas adsorption or catalysis [26,30,31], this strategy is designed to enhance interfacial compatibility with the polyamide layer and to exploit the photochromic and polarizable nature of SP for improved ion transport control.

In this study, MOF–808 was selected as the substrate material due to the following considerations: (i) the Zr clusters in the MOF–808 framework are coordinatively unsaturated and readily accessible for post-synthetic modifications; (ii) the pore size of MOF–808 (ca. 20 Å) is well-suited to accommodate small organic guest molecules; and (iii) MOF–808 exhibits excellent acid–base and thermal stability, ensuring its robustness in practical applications. To exploit these features, spiropyran (SP) molecules bearing terminal carboxyl groups were grafted onto MOF–808 through a post-synthetic modification strategy, yielding the functionalized material MOF–808–SP. The resulting MOF–808–SP was subsequently incorporated as a dopant into mixed-matrix membranes (MMMs), which were then evaluated for ion separation performance in both inorganic salt solutions and real seawater. Remarkably, the MOF–808–SP-based MMMs demonstrated a high ion separation efficiency that correlated positively with the MOF–808–SP loading while maintaining a stable ion retention rate. Furthermore, the membranes demonstrated outstanding operational stability, as evidenced by their ability to consistently sustain high NaCl rejection rates under continuous filtration for 24 h. More importantly, the MOF–808–SP MMMs exhibited excellent performance in desalination of seawater, underscoring their great potential for practical ion separation and seawater desalination applications.

## 2. Results and Discussion

### 2.1. Synthesis and Dosage Determination of Spiropyran (SP) Molecules

#### 2.1.1. Synthesis of SP Molecules

The synthetic procedure of SP molecules was described in the Supporting Materials (Scheme S1).

The improvement of surface properties in MOF–808 could be effectively achieved through the modification of SP molecules. Firstly, the original framework of MOF–808 was not destroyed due to the spiro ring structure in the SP molecules. Therefore, the original crystal form of MOF–808 could be well maintained. In addition, the carboxyl groups at both ends of the SP molecule could form relatively stable coordination bonds with unsaturated Zr6 clusters of MOF–808.

The carboxyl group exposed at the other end of SP molecules could also improve the surface properties of MOF–808. Thus, the SP molecules were the ideal candidates for the modifications of MOF–808.

#### 2.1.2. Determination of the Optimal Dosage of SP Molecules in the Modification Process

The optimal SP dosage was determined by 1H NMR analysis. The peak at 8.58 ppm was assigned to MOF–808 (Figure S1), while the signal at ~7.6 ppm was attributed to SP (Figure S2). By comparing the integrals of the characteristic peaks of SP and trimesic acid in the digested MOF–808–SP sample (Figure S3), we found that the integral ratios of trimesic acid to SP gradually decreased from 1:11.05 (SP dosage 6 mg), 1:8.05 (SP dosage 12 mg), to 1:7 (SP dosage 18 mg). This result indicates that the actual SP loading did not increase significantly when the SP dosage was raised from 12 mg to 18 mg. Therefore, an optimal dosage of 12 mg was selected for the preparation of MOF–808–SP.

### 2.2. Characterization of MOF–808–SP MMMs

#### 2.2.1. Characterization of MOF–808 and MOF–808–SP

The FTIR spectra of MOF–808 and MOF–808–SP are shown in Figure S4 [32]. For MOF–808–SP, two strong absorption peaks at 1089 and 1168 cm^−1^ can be attributed to the C–O stretching vibrations of the SP molecules. In addition, the N–C–O vibration of the SP moiety was observed at around 1750 cm^−1^, while the broad absorption band centered near 3000 cm^−1^ was assigned to the O–H stretching vibration of carboxyl groups in SP. The UV–vis spectra of the prepared MOFs were presented in Figure S5 [33]. In the solid–state spectra, pristine MOF–808 showed no significant absorption in the visible region, whereas MOF–808–SP exhibited a noticeable absorption band in the range of 350–600 nm, which is consistent with the characteristic red color of SP.

**Figure 1:**
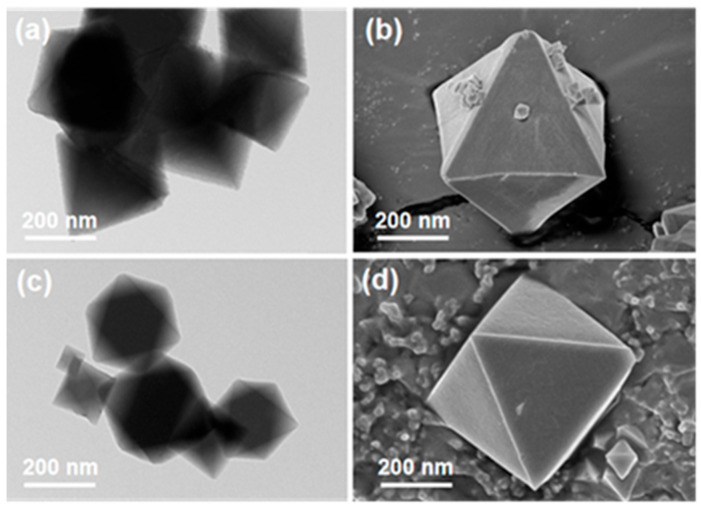
(**a**) TEM and (**b**) SEM of MOF–808; (**c**) TEM and (**d**) SEM of MOF–808–SP.

Transmission electron microscopy (TEM) and scanning electron microscopy (SEM) images of MOF–808 and MOF–808–SP were presented in Figure 1a–d, Figures S6 and S7. The MOF–808–SP shows a similar nanocrystal size (~200 nm) and octahedral morphology to the MOF–808 nanocrystal. It suggested the modification of SP molecules would not change the morphology and crystal size of MOF–808. 

**Figure 2:**
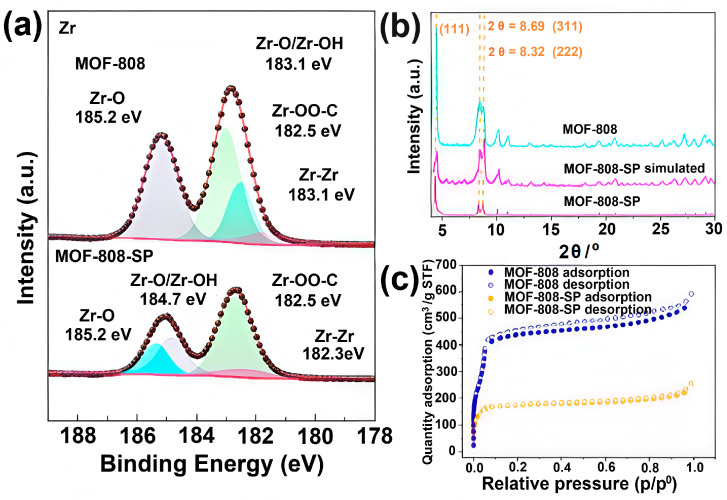
(**a**) Zr3d spectrum of MOF–808 and MOF–808–SP; (**b**) X-ray diffraction patterns of MOF–808–SP (pink) and MOF–808 (blue); (**c**) N_2_ adsorption–desorption curve of MOF–808–SP and MOF–808.

XPS characteristics of Zr 3d in MOF–808 and MOF–808–SP were displayed in Figure 2a [34,35,36]. The Zr–OOC peak intensity for MOF–808 was significantly enhanced compared to that for MOF–808–SP, while the peak intensity of Zr–O group is decreased. It might be attributed to the SP molecules being coordinated with the unsaturated Zr cluster in MOF–808. It confirmed that the O1s spectrum (Figure S9b) also showed that the decreased peak intensity of Zr–O, while the peak intensity of O–C=O at 533.7 eV increases after SP modification. It suggested the increased number of exposed COO– from the exposed carboxyl group in the SP molecule. As the C1s spectrum showed in Figure S9a, the higher C–O–C peak intensity of MOF–808–SP at 286.2 eV than that of MOF–808 was attributed to the C–O–C group in the SP molecule. Furthermore, the N1s spectrum (Figure S10) showed that MOF–808–SP had more evident sub-peaks of C–N and =N–H+ at 399.5 eV and 401.6 eV, respectively, corresponding to the tertiary amine in the SP molecule. The XPS, FTIR, and UV results confirmed the existence of SP molecules and the successful post-synthetic modification of SP molecules.

The powder X-ray diffraction (PXRD) patterns of MOF–808–SP, shown in Figure 2b and Figure S8, retained all characteristic reflections of pristine MOF–808, confirming the preservation of crystallinity and long-range order upon post-synthetic modification. The typical peaks of (311) and (222) planes of MOF–808 were located at 8.3° and 8.7°, respectively. Apart from minor intensity changes, these peaks did not change among the series of MOF–808–SP, indicating that adjusting the SP dosages did not drastically affect the crystal structure of MOF–808. However, a gradual decrease in the main diffraction peak intensity with increasing MOF–808–SP loading indicates that the incorporation of spiropyran molecules alters the electron density and partially fills the porous framework (Figures S12 and S13) [37]. XPS analysis further supports this interpretation, showing systematic shifts in the binding energies of Zr, O, C, and N species consistent with coordination of SP molecules to the unsaturated Zr clusters and the introduction of additional functional groups within the framework. Together, the PXRD and XPS results provide coherent evidence that the post-synthetic modification was successfully achieved at the molecular level while maintaining the structural integrity of the MOF–808 framework.

Figure 2c shows the N_2_ adsorption–desorption isotherms of the as-prepared MOFs. After SP modification, the N_2_ adsorption capacity of MOF–808 was significantly reduced, which could be attributed to the blocking of internal pores by SP molecules. The pore size distribution analysis further revealed that the pore volume of MOF–808–SP at 20 Å disappears, while a new pore volume emerged at 6 Å (Figure S11), indicating that the cavities of the MOF–808 framework were partitioned by SP molecules, consistent with the observed decrease in N_2_ adsorption capacity. Moreover, the disappearance of the 20 Å channel might also partially account for the enhanced ion retention performance observed below. These results align with the dynamic molecular sizes of folded (ca. 0.6 nm) and unfolded (ca. 1.8 nm) SP molecules that occupy the MOF–808 cavities and partition the internal channels upon post-synthetic modifications. Such pore occupation reduces surface area and pore size, supporting the XRD observations and explaining the improved ion separation performance through restricted ion transport while maintaining water permeability through well-integrated MOF–polymer interfaces [38,39].

#### 2.2.2. Characterization of Mixed Matrix Membrane

**Figure 3:**
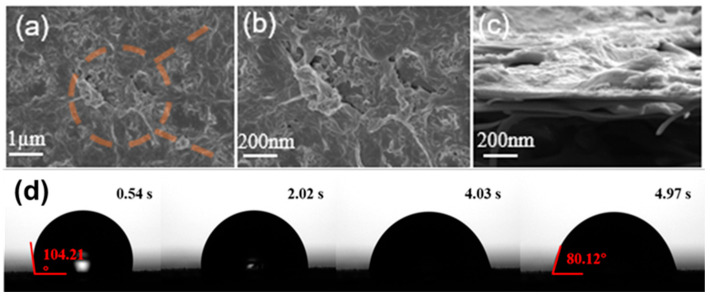
(**a**) and (**b**) Top- and (**c**) side views of the SEM of 808–SP–9 MMMs; (**d**) time-dependent water contact angles of 808–SP–9 MMMs.

Surface morphology of the mixed matrix membrane composed of MOF–808–SP, was analyzed through SEM (Figure 3a,b). The introduction of MOF–808–SP resulted in a special morphology on the surface of the membrane, indicating that the embedded nanoparticles have an impact on the crosslinking rate between the organic and liquid phases during the interfacial polymerization process. A cross-sectional view of the MOF–808–SP mixed matrix membrane (Figure 3c) revealed a dense film on the porous PES substrate, characterized by numerous fine granular protrusions on the membrane surface, accompanied by occasional larger protrusions. The surface roughness of the membrane could be attributed to the aggregation of MOF–808–SP nanoparticles. The morphology of the membrane surrounding each protrusion, with radially wrapped shapes, further confirmed the successful incorporation of MOF–808–SP nanoparticles into the mixed matrix film.

In Figure 3d, Figures S14 and S15, the water contact angle of the mixed matrix membrane was shown. The average water contact angle of the membrane surface gradually decreased from 92.27° (MOF–808–SP–3) to 80.12° (MOF–808–SP–9), indicating an enhancement in surface hydrophilicity with increasing SP modification. To gain deeper insight into this phenomenon, we performed Zeta potential measurements to investigate the surface charge of MOF–808–SP (Figure S16). The results revealed that the surface charge of MOF–808 increases from 33.33 mV (MOF–808) to 42.75 mV after SP modification. Consequently, the gradual reduction in the water contact angle on the membrane surface could be attributed to the increasing content of MOF–808–SP.

### 2.3. Membrane Filtration Performance Test

**Figure 4:**
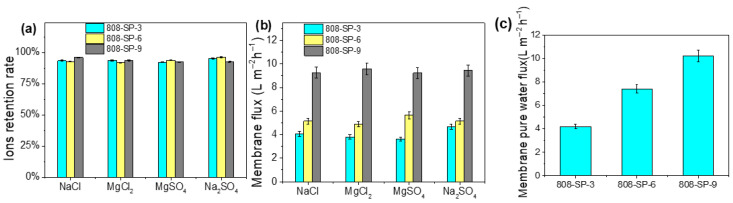
(**a**) Ion retention rate, (**b**) membrane flux, and (**c**) pure water flux of various MOF–808–SP MMMs.

MOF–808 MMMs were first prepared with varying amounts of MOF–808. As the MOF–808 loading increased to 9 mg, the ion rejection capacity of the membrane declined significantly (Figure S17), suggesting poor compatibility of MOF–808 with the membrane substrate. In contrast, the mixed matrix membranes prepared with different amounts of MOF–808–SP exhibited consistently stable ion retention performance (Figure 4). This indicated that embedding MOF–808–SP did not disrupt the compact polyamide structure, owing to the strong compatibility between MOF–808–SP and the polymer matrix. The enhanced compatibility could be ascribed to the exposed carboxyl groups of SPs anchored onto the Zr clusters of MOF–808, which increased the nucleophilicity of the MOF [37] and thereby reduced the formation of defects during film preparation. This SP modification enhances interfacial compatibility at the molecular level through coordination between the carboxyl groups of SP and the exposed Zr clusters in MOF–808, which promotes stronger chemical interactions with the polymer chains. These interactions effectively minimize interfacial nanogaps that typically arise during the incorporation of inorganic fillers, preserving the integrity of the polyamide selective layer. Consequently, water molecules preferentially permeate through the intrinsic MOF channels rather than through structural voids, leading to enhanced water flux while maintaining high ion rejection. In contrast, unmodified MOF–808 lacks such coordination sites, resulting in weaker interfacial adhesion and performance deterioration at higher loadings.

### 2.4. Membrane Stability Test

**Figure 5:**
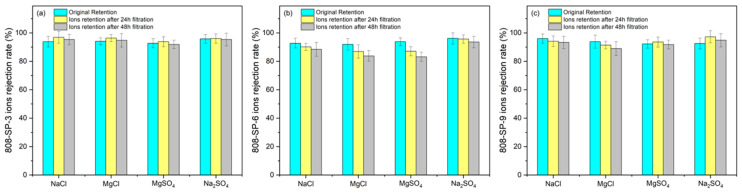
Changes in the ion rejection rate of (**a**) MOF–808–SP–3, (**b**) MOF–808–SP–6, and (**c**) MOF–808–SP–9 MMMs.

The long-term stability of the membranes was evaluated through continuous NaCl filtration over 48 h (Figure 5). For 808–SP–3, a slight increase in ion rejection was observed, suggesting that the overall membrane structure remained stable after extended operation. This improvement was likely attributed to concentration polarization at the membrane surface, where NaCl crystallite deposition partially blocked the pores. In contrast, when the MOF–808–SP loading was increased to 6 mg, a slight decrease in ion rejection after 48 h indicated the presence of defects in the selective separation layer of the mixed matrix membrane, as further confirmed by SEM analysis (Figure S18). Among the tested inorganic salts, MgSO_4_ exhibited the most pronounced decrease in ion rejection, dropping from 93.61% to 87.07%. Notably, when the MOF–808–SP loading was increased to 9 mg, the ion retention remained essentially unchanged after 24 h of NaCl filtration, demonstrating excellent membrane stability.

**Figure 6:**
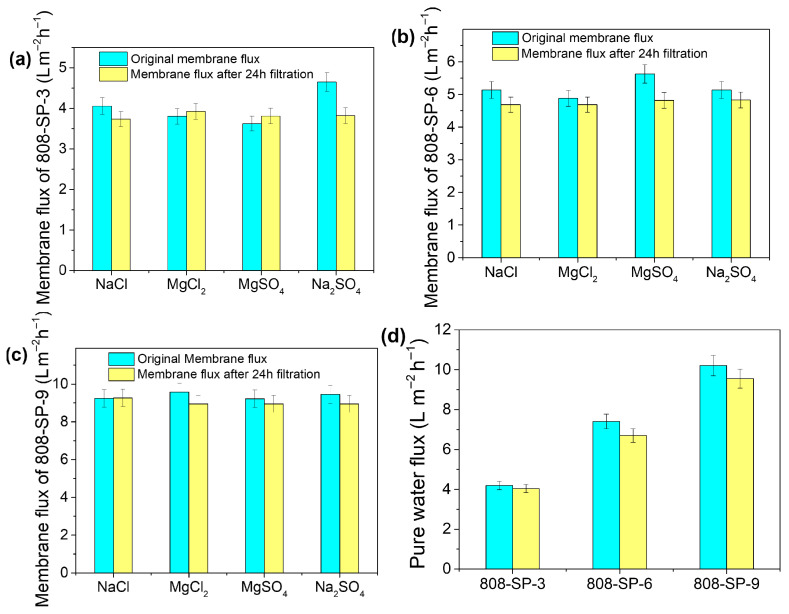
Changes in the membrane flux of (**a**) MOF–808–SP–3, (**b**) MOF–808–SP–6, and (**c**) MOF–808–SP–9, and (**d**) variation in pure water flux.

The membrane flux and pure water flux of three mixed matrix membranes were tested after filtering NaCl solution for 48 h, as indicated in Figure 6. Generally, the membrane flux of the mixed matrix membrane showed decline towards various inorganic salt solutions after 48 h operation. It may be attributed to the precipitation of the NaCl particles on the membrane surface due to long-term high operating pressure filtration. The NaCl particles would block the membrane pores and increase the mass transfer resistance of water molecules and thus led to the reduction in membrane flux and pure water flux [40,41]. Furthermore, the increase in membrane flux was found to positively correlate with MOFs dosage. This was due to the higher material loading of the membrane will provide more pores on the surface, leading to an increased number of water molecule transport channels on the membrane surface.

Notably, the membrane fluxes of 808–SP–3 and 808–SP–6 were lower than that of the pristine polyamide membrane, indicating that the modification of MOF–808 with SP enhanced the nucleophilicity of its surface and led to the formation of a denser mixed matrix membrane during film formation [37,39]. In contrast, 808–SP–9 exhibited an increased membrane flux while maintaining a similar ion rejection rate. This suggested that the incorporation of MOF–808–SP introduced additional transport channels for water molecules, thereby enhancing the flux of the SP-based mixed matrix membranes. Thus, as the MOF–808–SP loading increases, the number of water transport channels in the mixed matrix membrane also increases, resulting in higher water flux.

### 2.5. Seawater Treatment Performance

Combining the single inorganic salt solution tests and stability analysis, the 808–SP–9 was employed to study the membrane filtration performances of seawater. The initial ion rejection rate of the membrane in seawater (86.13%) was inferior to that in the single inorganic salt solution filtration test. The MgSO_4_ solution accounted for the lowest rejection rate at 92.28%. The membrane flux (3.92 L m^–2^ h^–1^) with seawater is 1.78 times lower than that of pure water (10.2 L m^–2^ h^–1^), which was likely attributed to the complex composition of seawater. The seawater used in this study contained typical ions such as Na^+^, K^+^, Mg^2+^, Ca^2+^, Al^3+^, Cl^−^, SO_4_^2−^, and HCO_3_*^−^*, whose interactions and precipitation tendencies can influence membrane fouling and transport behavior.

**Figure 7:**
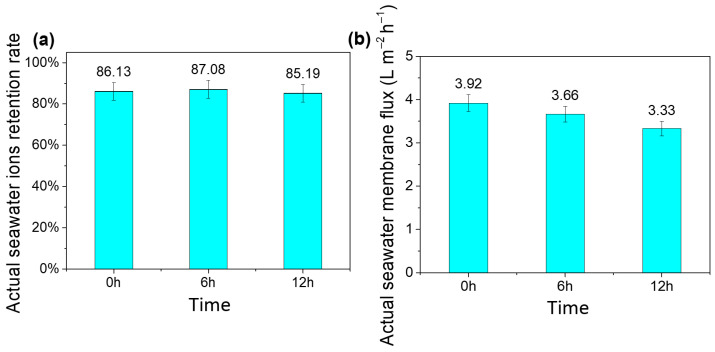
(**a**) Ion rejection rate and (**b**) water flux in the system of seawater.

The stability of a mixed matrix membrane was assessed using seawater (Figure 7a,b). Within the first 6 h of continuous operation, the membrane exhibited an increased ion retention rate of 87.09% while the membrane flux decreased to 3.66 L m^–2^ h^–1^. It demonstrated that the clogging of membrane channels and the selective separation layer remained. However, after 12 h of continuous operation, both the ion rejection rate and membrane flux decreased, indicating the selective separation layer was undermined. This decline can be attributed to fouling caused by the precipitation of multivalent salts such as CaCO_3_ and MgCO_3_, as well as partial organic deposition, which blocks membrane pores and gradually deteriorate the selective layer. In the long term, in a high-pressure environment, inorganic mineral ions such as calcium, magnesium, carbonate, sulfate, and phosphate in seawater would precipitate from the solution near the membrane surface, causing a reduction in membrane flux due to the blockage of membrane pores [42,43]. Moreover, these precipitated inorganic mineral ions would gradually damage the selective separation layer of the mixed matrix membrane, leading to a decline in both the rejection rate and membrane flux. Compared with other reported MOF-based MMMs (e.g., MIL–101 (Cr) and UiO–66–NH_2_), the MOF–808–SP membrane exhibits competitive desalination performance with stable ion retention, highlighting the beneficial role of SP modification in improving interface stability and fouling resistance.

## 3. Experimental

### 3.1. Materials

Unless otherwise mentioned, all reagents and solvents were purchased from commercial sources and used as received without further purification.

### 3.2. Syntheses

#### 3.2.1. Synthesis of Spiropyran (SP)

Syntheses of organic precursors for SP were detailed in the Supporting Information.

2,3,3–tetramethylspiro–3H–Indole–5–carboxylic acid (1.00 g, 4.5 mmol) and 3–formyl–4–hydroxybenzoic acid (0.53 g, 3.1 mmol) were dissolved in ethanol (100 mL) in a round-bottom flask equipped with a reflux condenser. Under a nitrogen atmosphere, triethylamine (1.1 mL) was added dropwise using a precision injector, and the mixture was refluxed at 80 °C for 8 h. After the reaction was completed, 0.1 M HCl was added dropwise to the reaction mixture until a precipitate formed. The resulting solid was collected by filtration, washed with water (3 × 20 mL), and dried at 60 °C. The obtained red powder was then re-dissolved in 0.1 M KOH (20 mL), and 0.1 M HCl was added dropwise until precipitation occurred again. The solid was collected, washed with water (3 × 20 mL), and dried at 80 °C to yield the product SP as a red powder (410 mg, 1.12 mmol, 36%) (Figures S1–S3).

#### 3.2.2. Synthesis of MOF–808

MOF–808 was synthesized following a reported procedure [44] with minor modifications. ZrCl_4_ (48 mg, 0.20 mmol) and trimesic acid (33 mg, 0.16 mmol) were dissolved in DMF (6 mL) and formic acid (6 mL) in a 25 mL flask under ultrasonication for 5 min to obtain a clear solution. The flask was sealed and heated at 120 °C for 48 h. The resulting solid was collected by centrifugation, washed sequentially with DMF and acetone, and dried at 60 °C to afford MOF–808 as a white powder.

#### 3.2.3. Synthesis of MOF–808–SP

A total of 10 mg of MOF–808 and 12 mg SP were added to 2 mL DMF and placed in an oven at 70 °C for 48 h. After reaction, the resultant red solid was separated by filtration, washed three times with DMF and absolute ethanol, and dried in an oven at 80 °C, to yield MOF–808–SP.

### 3.3. Fabrication of MOF–808–SP Mixed Matrix Membranes (MMMs)

**Scheme 1:**
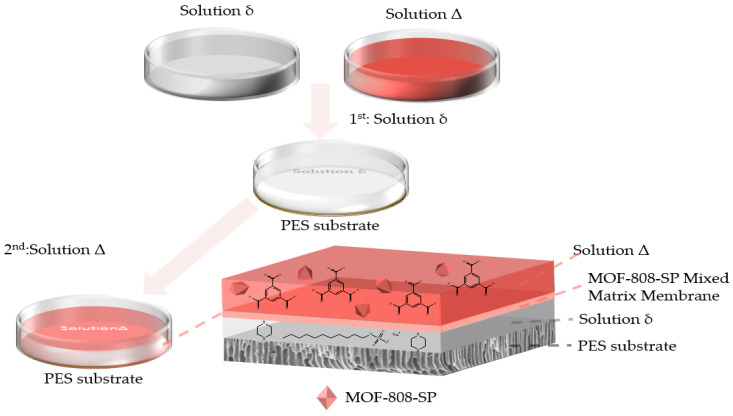
Schematic diagram of a representative MOF–808–SP mixed matrix membrane.

MOF–808–SP MMMs were prepared by the interfacial polymerization method as illustrated in Scheme 1. Piperazine (PIP, 60 mg, 0.60 mmol) and sodium dodecyl sulfate (SDS, 5 mg, 0.002 mmol) were dissolved in deionized water (20 mL) to form solution δ. Trimesoyl chloride (TMC, 10 mg, 0.003 mmol) and MOF–808–SP-n (*n* = 3, 6, or 9 mg) were dispersed in n–hexane (10 mL) under ultrasonication for 30 s to afford solution Δ. A polyethersulfone (PES) substrate placed in a Petri dish was first immersed in solution δ for 3 min. Excess solution was removed, and solution Δ was then added and allowed to react for 1 min. The excess organic solution was decanted, and the membrane was rinsed three times with n–hexane to remove unreacted TMC. Finally, the membrane was heated at 80 °C for 7 min to strengthen the interfacial polymerization, yielding MOF–808–SP–n MMMs (*n* = 3, 6, 9 mg; see Figure S15).

### 3.4. Characterizations

1H–NMR spectra were recorded on a Bruker AVANCE III 400MHz spectrometer (Bruker Co., Ettlingen, Germany). FTIR spectra recorded by Fourier transform infrared spectroscopy (Nicolet IS10, Thermo Fisher Scientific, Waltham, MA, USA). PXRD was performed on a Rigaku Miniflex 600 Benchtop X-ray diffraction instrument (Rigaku Co., Tokyo Metropolis, Japan) using Cu Kα radiation (λ = 1.5418 Å) operated at 40 kV and 40 mA. Samples were prepared as flat powder films on low-background sample holders. Data were collected over a 2θ range of 2.0° to 30.0°, with a step size of 0.02° and a counting time of 1 s per step. UV–vis absorbance spectra of liquid samples were collected at room temperature on a Shimadzu UV–2550 spectrophotometer, and those of solid samples were collected at room temperature on the Shimadzu UV–2550 spectrophotometer (Shimadzu Co., Osaka City, Japan) equipped with Labsphere integrating over the spectral range λ = 200–800 nm using the BaSO_4_ as standards. SEM was performed on JSM6700–F Field Emission Scanning Electron Microscope (JEOL Ltd., Akishima-shi, Japan) and scanning electron microscope (Gemini–SEM 300–71–11, Carl Zeiss AG, Oberkochen, Germany). Nitrogen adsorption–desorption isotherms were measured at 77 K on a Micromeritics ASAP 2460 analyzer to determine Brunauer–Emmett–Teller (BET) surface areas and pore volumes (Micromeritics Instr. Co., Norcross, GA, USA). Zeta potential measurements were carried out using a 90Plus PALS particle size and zeta potential analyzer (Brookhaven Co., Holtsville, NY, USA). The surface hydrophilicity/hydrophobicity of MMMs was evaluated by water contact angle measurements using a Theta Flex analyzer (Biolin Scientific AB, Gothenburg, Sweden).

### 3.5. Membrane Filtration Performance Test

The filtration performance of the prepared membranes was tested using a fuel separation device (Nanjing Hope Analysis Equipment Co., Ltd., Nanjing, China) with an effective membrane area of 16 cm^2^. The performance of nanofiltration membranes was assessed using three key indicators: membrane flux, pure water flux, and rejection rate.

(1) Membrane flux (Q) was calculated according to the following equation:

$$Q=\frac{\Delta V}{S\Delta T\Delta P}$$

where ΔV is the permeate volume (L), S is the effective membrane area (m^2^), ΔT is the filtration time (h), and ΔP is the pressure exerted on the membrane surface (MPa). When the filtrate is pure water, the calculated membrane flux is the pure water flux.

(2) Rejection rate (R) was calculated using the following equation:

$$R=\left(1-\frac{C_{f}}{C_{0}}\right)\times 100\%$$

where C_f_ is the solute concentration of the filtrate in the permeate and C_0_ is the concentration of filtrate in the original solution. In this study, Na_2_SO_4_, MgSO_4_, MgCl_2_, and NaCl were selected as representative inorganic salts, each prepared at a concentration of 1000 mg L^–1^. In addition, the concentration of inorganic salt, the values of C_f_ and C_0_ were determined from conductivity measurements using a conductivity meter.

### 3.6. Membrane Stability Test

The stability of the MMMs was evaluated by monitoring the ion rejection performance over time. The initial rejection rate of the membrane toward a salt solution was recorded as R_0_. The membrane was then subjected to continuous filtration of a NaCl solution for 24 h at 0.6 MPa and a flow rate of 15 mL min^–1^. Subsequently, different salt solutions were filtered sequentially, and the corresponding rejection rate was recorded as R_24_. Membrane stability was assessed by comparing the difference between R_24_ and R_0_.

In trials of seawter, ion concentrations were determined to be Na^+^ (9623 mg L^−1^), K^+^ (323 mg L^−1^), Mg^2+^ (1068 mg L^−1^), Ca^2+^ (339 mg L^−1^), Al^3+^ (9623 mg L^−1^), Cl^-^ (15,743 mg L^−1^), SO_4_^2--^ (2734 mg L^−1^), HCO_3_^-^ (116 mg L^−1^).

## 4. Conclusions

In this work, a pure spiropyran (SP) molecule was successfully synthesized and characterized by ^13^C NMR and ^1^H NMR analyses. XRD and XPS confirmed that SP was anchored onto MOF–808 (denoted as MOF–808–SP), effectively modulating the surface properties of the MOF. Incorporation of MOF–808–SP into mixed matrix membranes resulted in a proportional increase in membrane flux with increasing filler loading, while maintaining high ion retention, demonstrating improved compatibility between MOF–808–SP and the polymer substrate. Among the fabricated membranes, 808–SP–9 exhibited a high ion rejection rate (92%) and a 2.44-fold enhancement in pure water flux (10.21 L m^−2^ h^−1^) compared with 808–SP–3. Continuous filtration of NaCl solution for 24 h showed stable performance, highlighting the membrane’s operational stability under short-term testing. Post-synthetic SP modification of MOF–808 strengthens the MOF–polymer interface, allowing simultaneous improvement of water flux and ion rejection by maintaining the structural integrity of the selective layer. This molecular-level enhancement helps overcome the traditional trade-off between permeability and selectivity in polymer-based membranes.

While these results are promising, long-term performance, fouling resistance, and the potential degradation of the SP functionality under extended operation remain to be fully assessed. Furthermore, considerations such as scalability and the practical integration of MOF–SP membranes into large-scale water treatment systems require further investigation. Overall, MOF–808–SP provides a viable platform for designing advanced mixed matrix membranes with balanced permeability and selectivity, pending further validation under real-world desalination and wastewater treatment conditions.

## Data Availability

The original contributions presented in this study are included in the article/Supplementary Material. Further inquiries can be directed to the corresponding author(s).

## Supplementary Materials

The following supporting information can be downloaded at: https://www.mdpi.com/article/10.3390/molecules30234554/s1, Scheme S1: Synthetic route of the spiropyran molecule; Figure S1: ^1^H NMR after digestion of MOF–808; Figure S2: ^1^H NMR of MOF–808–SP after digestion and ^1^H NMR of SP molecules; Figure S3: ^1^H NMR of MOF–808–SP prepared by different SP additions; Figure S4: Fourier transform infrared spectrum (FTIR) of MOF–808 and MOF–808–SP; Figure S5: UV-Vis of MOF–808 and MOF–808–SP; Figure S6: TEM of MOF–808 and MOF–808–SP; Figure S7: SEM of MOF–808 and MOF–808–SP; Figure S8: PXRD of MOF–808–SP prepared by different SP dosages; Figure S9: (a) O1s XPS spectrum (b) C1s XPS spectrum of MOF–808 and MOF–808–SP; Figure S10: N1s XPS spectrum; Figure S11: pore size analysis of MOF–808 and MOF–808–SP; Figure S12: As-prepared MOF–808(-SP) mixed matrix membrane; Figure S13: X-ray diffraction pattern (XRD) of MOF–808–SP mixed matrix membrane; Figure S14: Water contact angle of mixed matrix membrane of 808–SP (a) 808–SP-3 (b) 808–SP-6 (c) 808–SP-9; Figure S15: (a) Trend of water contact angle (b) Average water contact angle of three mixed matrix membranes; Figure S16: DLS and PALS of MOF–808 and MOF–808–SP; Figure S17: Ions retention of mixed-matrix membranes fabricated with MOF–808; Figure S18: SEM image of Mixed-matrix membranes fabricated with MOF–808–SP (a) 808–SP-3 (b) 808–SP-6 (c) 808–SP-9; Figure S19: ^1^H NMR of 2,3,3-trimethyl-3H-indole-5-carboxylic acid; Figure S20: ^1^H NMR of 1,2,3,3-Tetramethyl-3H-indole-5-carboxylic acid; Figure S21: ^1^H NMR of 3-formyl-4-hydroxybenzoic acid.
