# Supplementary material for: Post–Synthetic Modification of MOF–808 for Mixed Matrix Membranes with High and Stable Ion Separation Capacity
Source: Molecules. 2025 Nov 26;30(23):4554. doi: 10.3390/molecules30234554 (PMC12693105; doi:10.3390/molecules30234554)
Supplement: Supplementary file 1 [file molecules-30-04554-s001.zip › molecules-3980884-supplementary.pdf]

Supporting information for

Post-Synthetic Modification of MOF-808 for mixed matrix membranes with high and stable ion separation capacity

Bahar Karadeniz [1,2], Han-Liang Fang [1], Yi-Ying He [1], Qi-Lin Ye [1], Jun-Yu Chen [1*] and Jian Lü[1,3*]

1 Fujian Provincial Key Laboratory of Soil Environmental Health and Regulation, College of Resources and Environment, Fujian Agriculture and Forestry University, Fuzhou 350002, China.

2 Ruđer Bošković Institute, Bijenička cesta 54, 10000 Zagreb, Croatia.

3 FAFU-DAL Joint College (International College), Fujian Agriculture and Forestry University, Fuzhou 350108, China.

[*] Correspondence: chenjunyu21222@163.com (J. C.); jian_lu_fafu@163.com (J. L.)

Test S1. Synthesis of 2,3,3-trimethyl-3H-indole-5-carboxylic acid

p-Hydrazinobenzoic acid (1.5 g, 9.85 mmol) and 3-methyl-2-butanone (1.27 g, 14.79 mmol) were added to anhydrous acetic acid (20 mL), and the resulting mixture was stirred at 120 °C for 30 h. After the reaction was completed, silica gel (approximately half the mass of the reaction mixture) was added to the obtained solution, and the solvent was removed under reduced pressure to yield a solid residue. The resulting solid was subjected to silica gel column chromatography using CH_2Cl_2/CH_3CN (10:1, v/v) containing 0.5% triethylamine as the eluent. Fractions containing the desired product were collected and evaporated under reduced pressure. The residue was dried under vacuum at 80 °C for 24 h to give the product as a solid (1.05 g, 5.1 mmol, 51.7% yield). ^{1}H NMR (DMSO-d6), δ: 12.77 (s), 7.96-7.94 (m), 7.88-7.85 (m), 7.46 (d, J = 8.0 Hz), 2.21 (s), 1.23 (s).

Test S2. Synthesis of 1,2,3,3-Tetramethyl-3H-indole-5-carboxylic acid

3,3-Trimethyl-3H-indole-5-carboxylic acid (1.0 g, 4.9 mmol) was dissolved in acetonitrile (75 mL) in a round-bottom flask equipped with a reflux condenser. Under a nitrogen atmosphere, methyl iodide (2 mL) was added dropwise using a syringe, and the reaction mixture was stirred at 80 °C for 30 h. Upon completion, the resulting yellow solid was collected and washed with acetonitrile until the filtrate became light yellow or nearly colorless. n-Hexane was then added to the residue, resulting in the formation of an agglomerated yellow solid. The mixture was shaken vigorously to slightly disperse the solid and allowed to stand for 30 min; this process was repeated three times. After removing n-hexane from the final washing step, the solid was dried under vacuum at 80 °C for 12 h to afford the desired product as a yellow solid (512 mg, 2.3 mmol, 46.9% yield). ^{1}H NMR(DMSO-d_6) δ:8.39 (s), 8.20 (d, J = 8.0 Hz), 8.02 (d, J = 8.4 Hz), 4.00 (d, J = 9.1 Hz), 2.80 (s), 1.57 (s).

Test S3. Synthesis of 3-Formyl-4-hydroxybenzoic acid

p-Hydroxybenzoic acid (3.0 g, 21.7 mmol) was dissolved in trifluoroacetic acid (15 mL) to give solution θ. Hexamethylenetetramine (urotropine, 3.1 g, 21.4 mmol) was dissolved in trifluoroacetic acid (10 mL) to give solution γ. Solution θ was transferred to a 100 mL round-bottom flask equipped with a reflux condenser, and under a nitrogen atmosphere, solution γ was added dropwise using a 5 mL syringe. The mixture was stirred at 90 °C for 3 h. After completion of the reaction, a transparent pale-yellow solution was obtained. Pre-prepared 4 M HCl solution was added, and the mixture was stirred at room temperature for 3 h. During this process, a solid gradually precipitated, changing in color from white to light yellow. The resulting solid was collected by filtration, washed with water three times, and dried under vacuum at 80 °C for 12 h to afford the product as a light-yellow solid (830 mg, 5.0 mmol, 23% yield).

Test S4. Determination of the SP dosage

To confirm the successful incorporation of SP molecules into MOF-808, ^{1}H NMR analysis was performed on digested samples. Specifically, 3 mg of MOF-808-SP was digested, and the resulting solution was compared with the ^{1}H NMR spectrum of the pure SP molecule to identify the characteristic peaks of SP in MOF-808-SP. Similarly, 3 mg of pristine MOF-808 was digested with the addition of excess trimesic acid to determine the characteristic peaks of trimesic acid. MOF-808-SP samples were prepared using different initial amounts of SP, and the integral ratios of the characteristic ^{1}H NMR peaks corresponding to trimesic acid and SP were analyzed. As the SP dosage increased, the integral ratio of trimesic acid to SP progressively decreased, with ratios of 1:11, 1:8, and 1:7 for SP dosages of 6, 12, and 18 mg, respectively. Since the actual SP loading showed negligible variation between the 12 mg and 18 mg SP dosages, the 12 mg SP dosage was selected as the optimal condition for the preparation of MOF-808-SP.

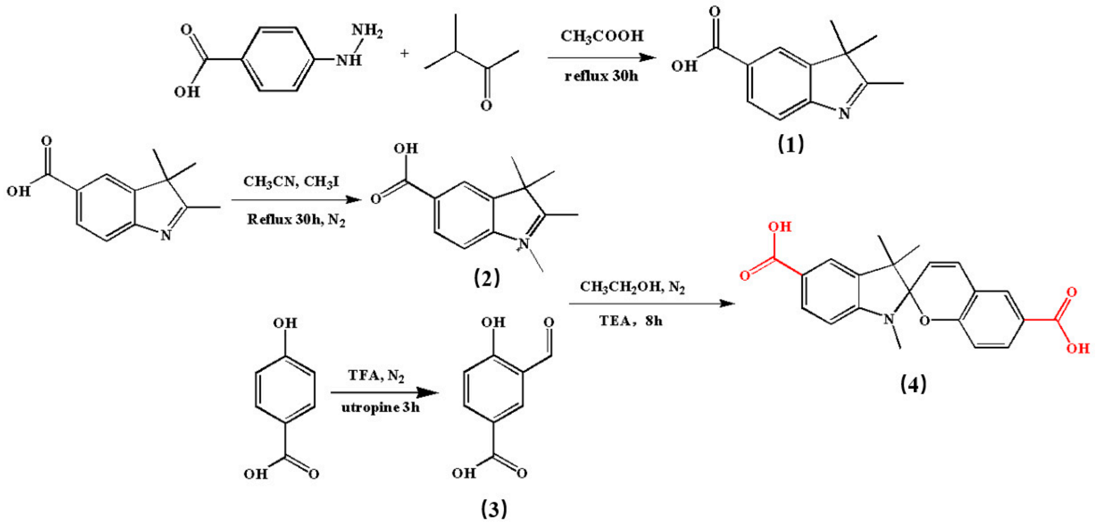

Scheme S1 Synthetic route of the spiropyran molecule.

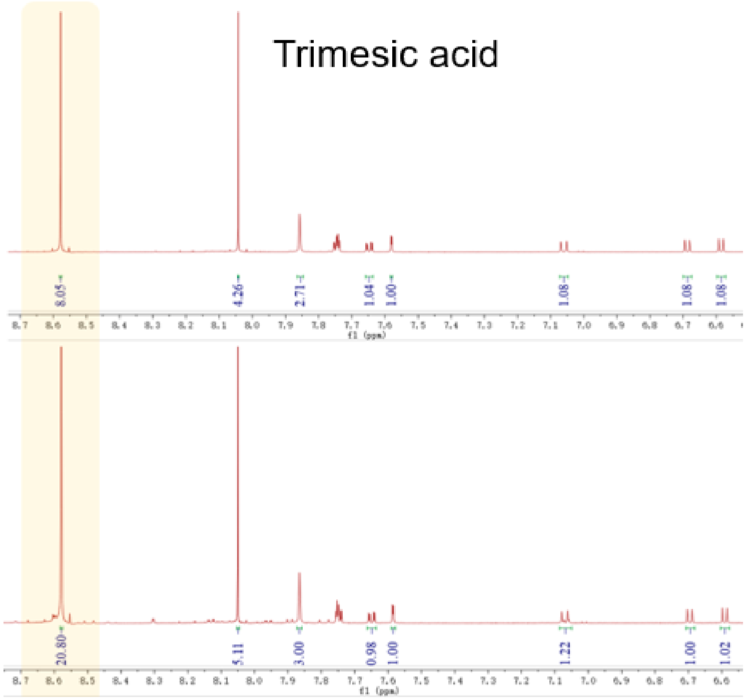

Figure S1 ^{1}H NMR after digestion of MOF-808.

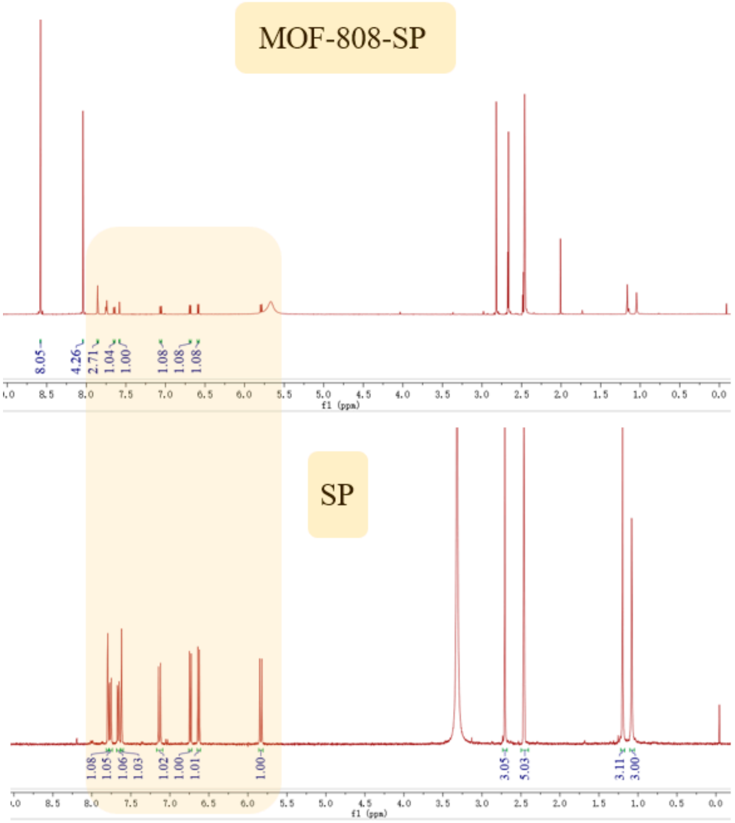

Figure S2 ^{1}H NMR of MOF-808-SP after digestion and ^{1}H NMR of SP molecules.

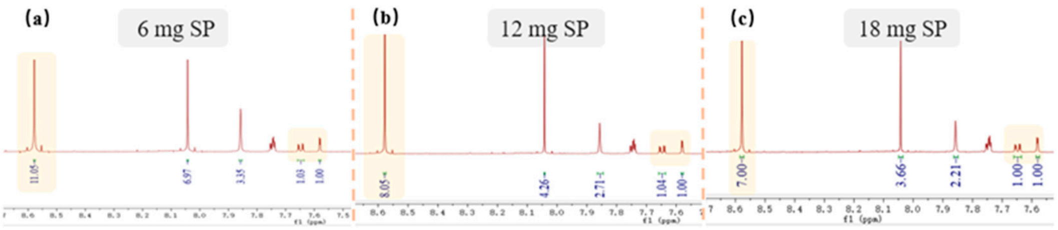

Figure S3 ^{1}H NMR of MOF-808-SP prepared by different SP additions.

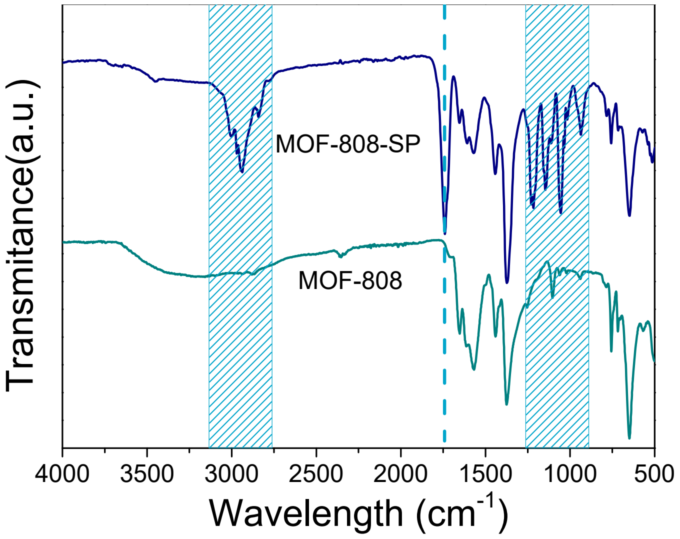

Figure S4 Fourier transform infrared spectrum (FTIR) of MOF-808 and MOF-808-SP.

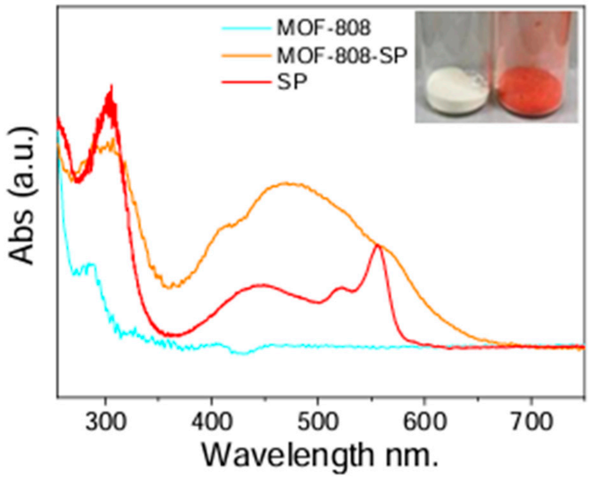

Figure S5 UV-Vis of MOF-808 and MOF-808-SP.

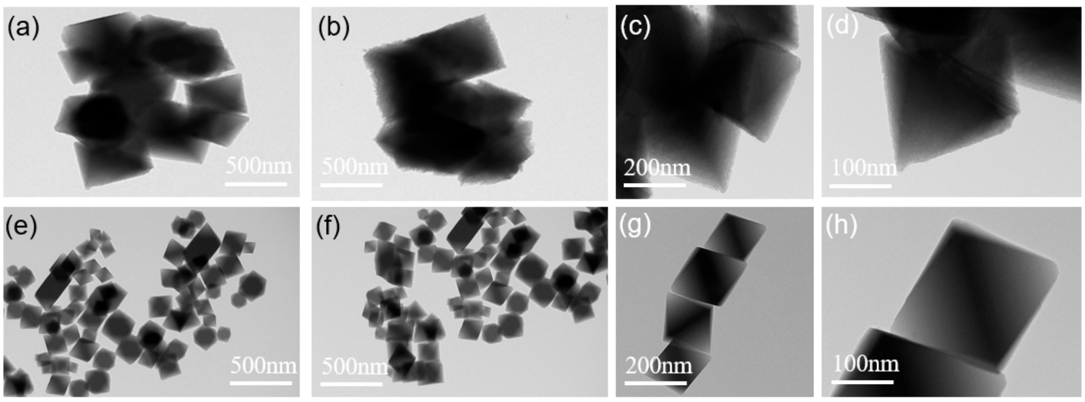

Figure S6 TEM of MOF-808 and MOF-808-SP.

(a) (b) (c) (d) MOF-808 (e) (f) (g) (h) MOF-808-SP.

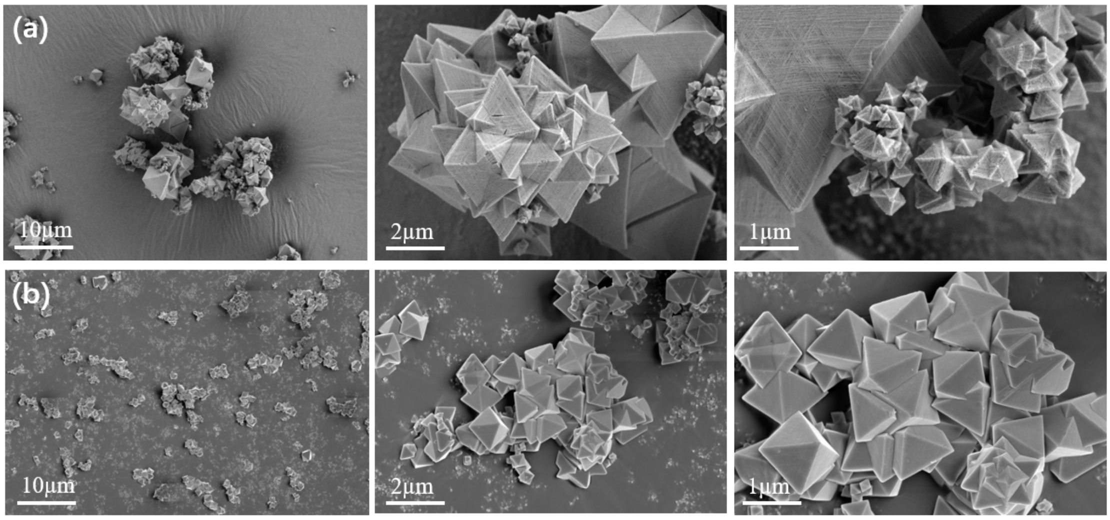

Figure S7 SEM of MOF-808 and MOF-808-SP.

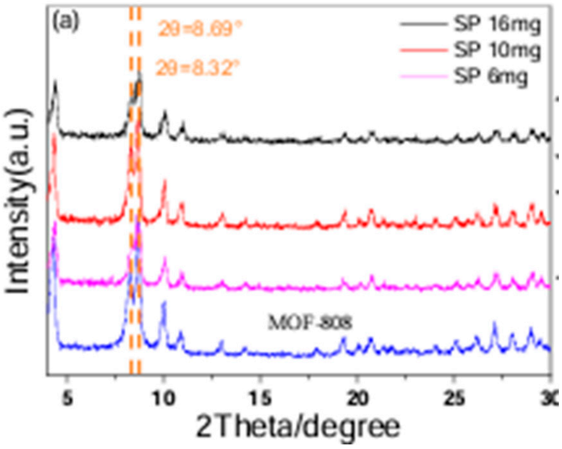

Figure S8 PXRD of MOF-808-SP prepared by different SP dosages.

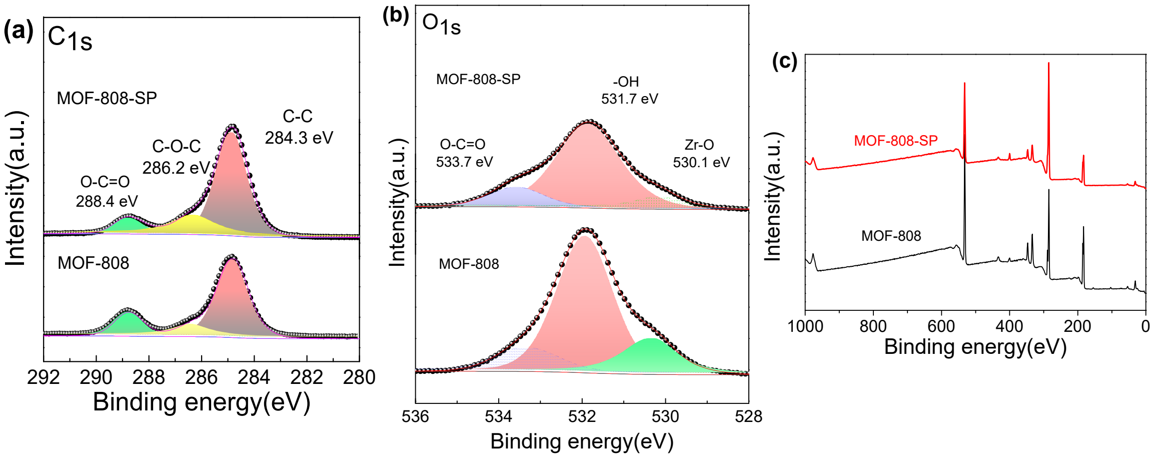

Figure S9 (a) O1s XPS spectrum (b) C1s XPS spectrum of MOF-808 and MOF-808-SP.

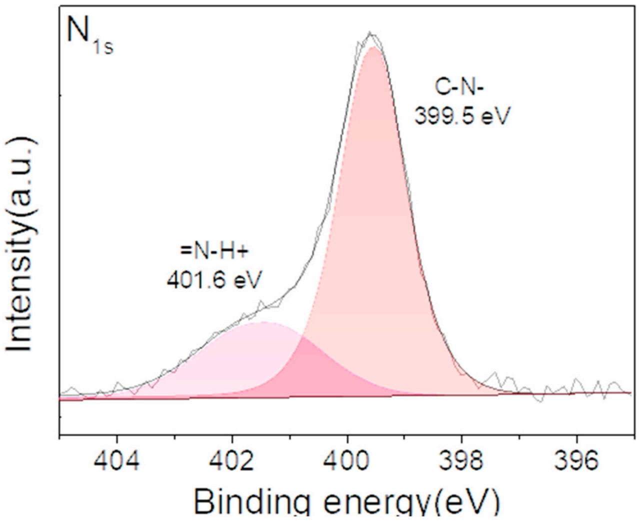

Figure S10 N1s XPS spectrum.

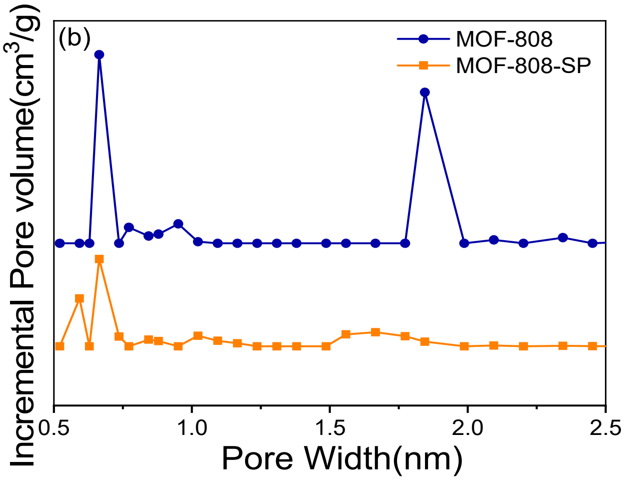

Figure S11 pore size analysis of MOF-808 and MOF-808-SP.

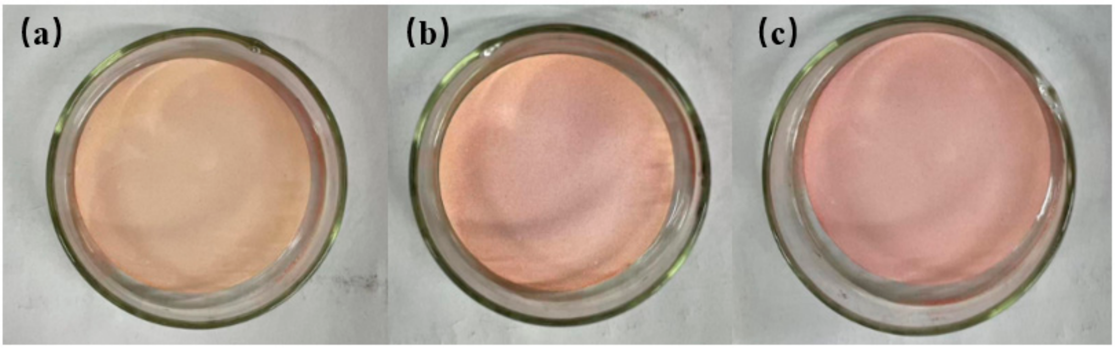

Figure S12 As-prepared MOF-808(-SP) mixed matrix membrane.

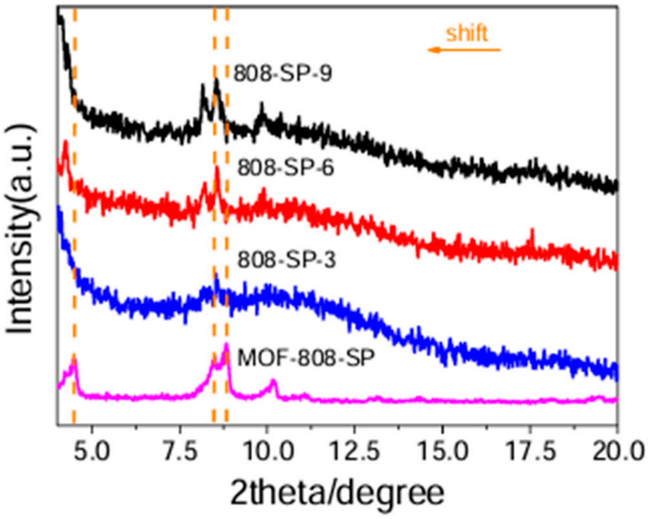

Figure S13 X-ray diffraction pattern (XRD) of MOF-808-SP mixed matrix membrane.

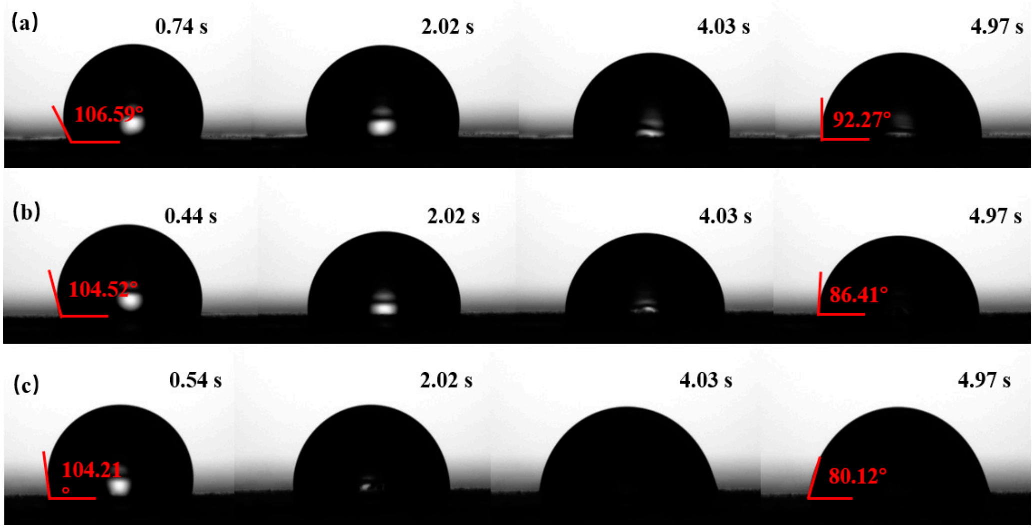

Figure S14 Water contact angle of mixed matrix membrane of 808-SP (a) 808-SP-3 (b) 808-SP-6 (c) 808-SP-9.

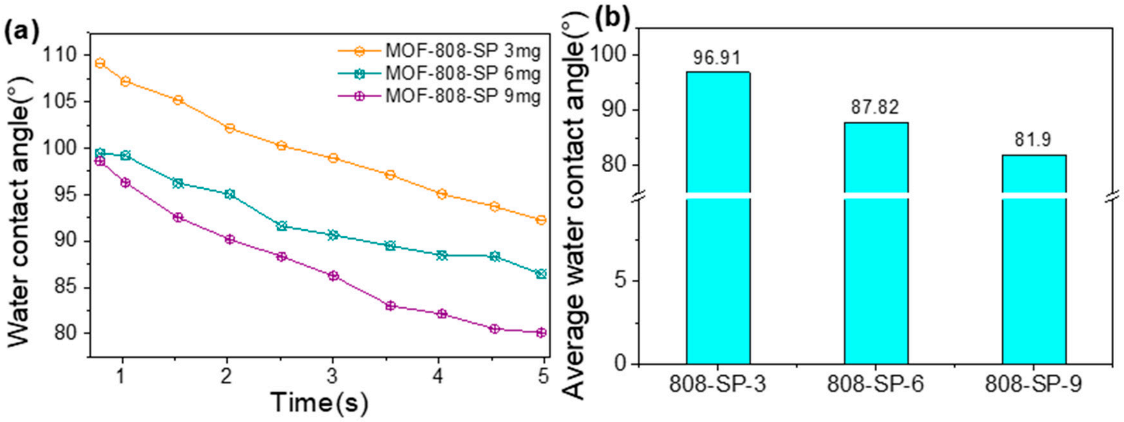

Figure S15 (a) Trend of water contact angle (b) Average water contact angle of three mixed matrix membranes.

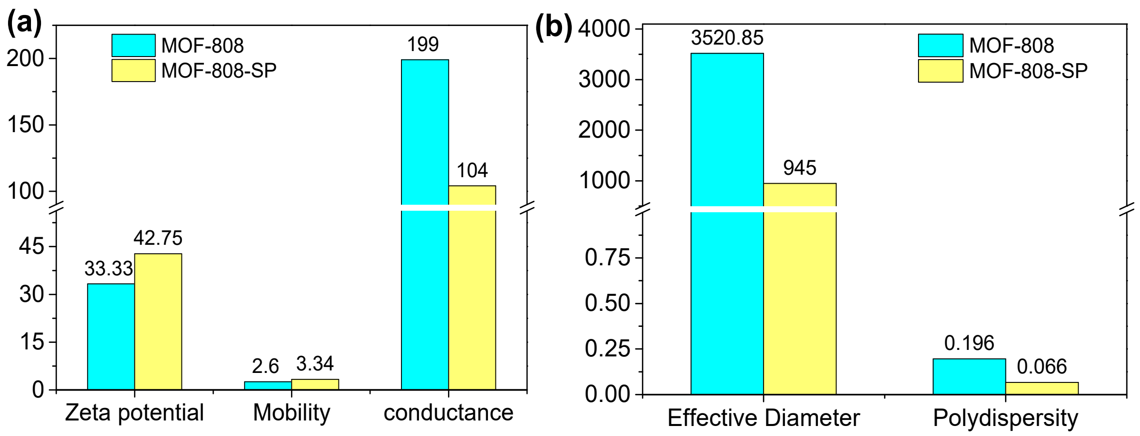

Figure S16 DLS and PALS of MOF-808 and MOF-808-SP.

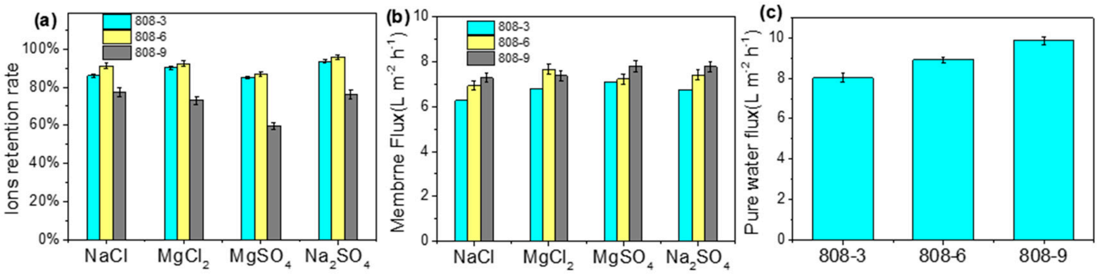

Figure S17 Ions retention of mixed-matrix membranes fabricated with MOF-808.

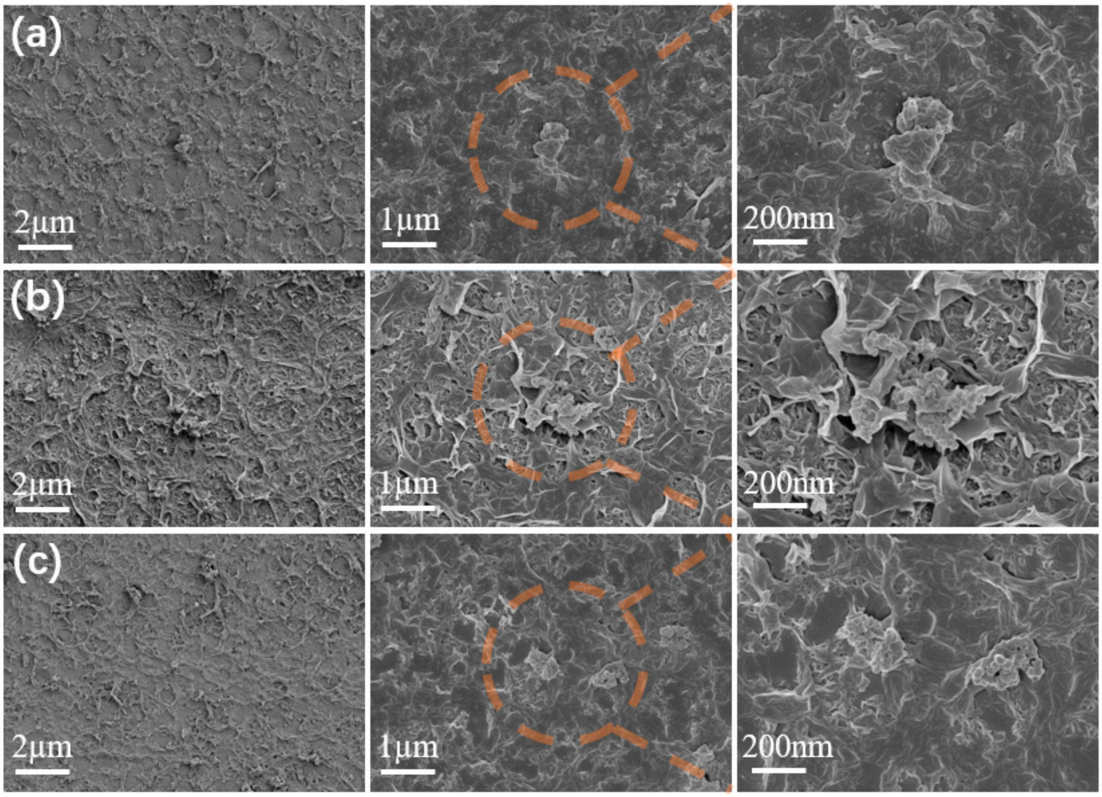

Figure S18 SEM image of Mixed-matrix membranes fabricated with MOF-808-SP (a) 808-SP-3 (b) 808-SP-6 (c) 808-SP-9.

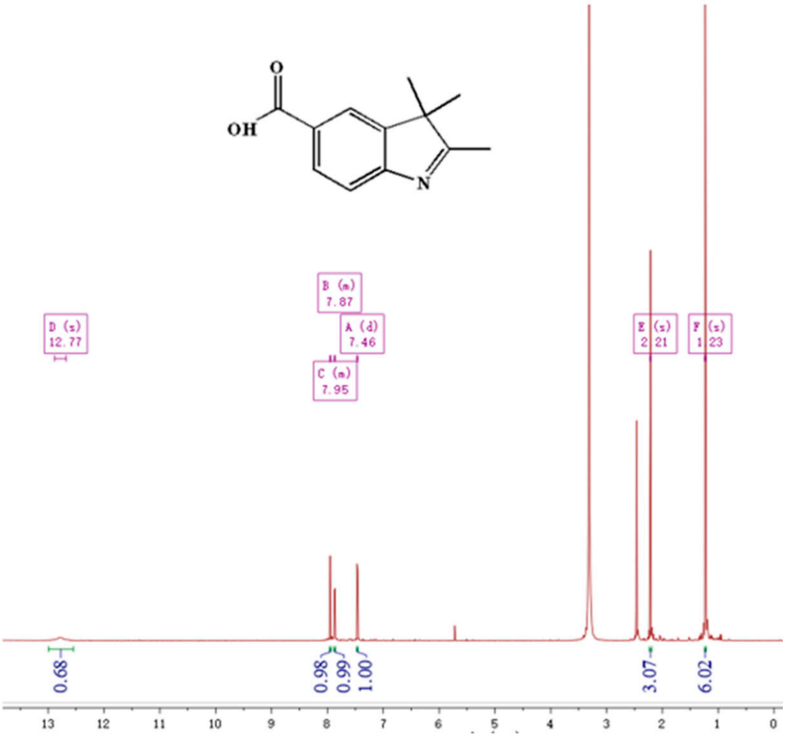

Figure S19 ^{1}H NMR of 2,3,3-trimethyl-3H-indole-5-carboxylic acid.

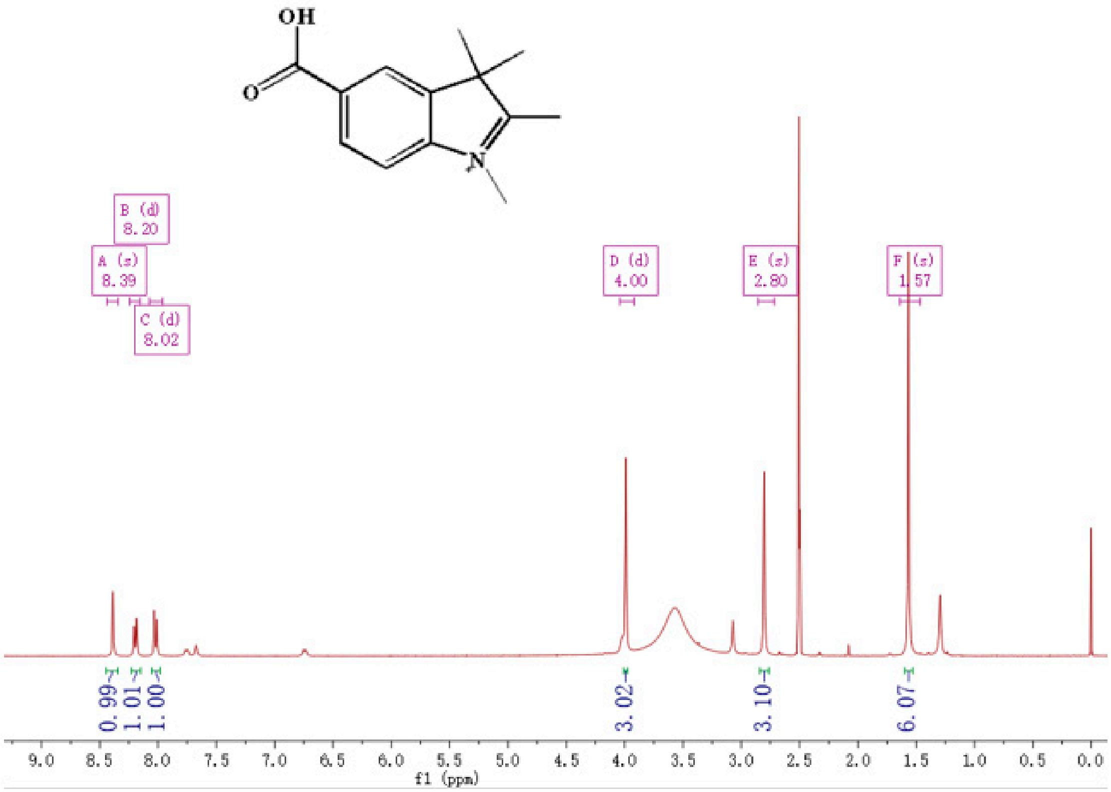

Figure S20 ^{1}H NMR of 1,2,3,3-Tetramethyl-3H-indole-5-carboxylic acid.

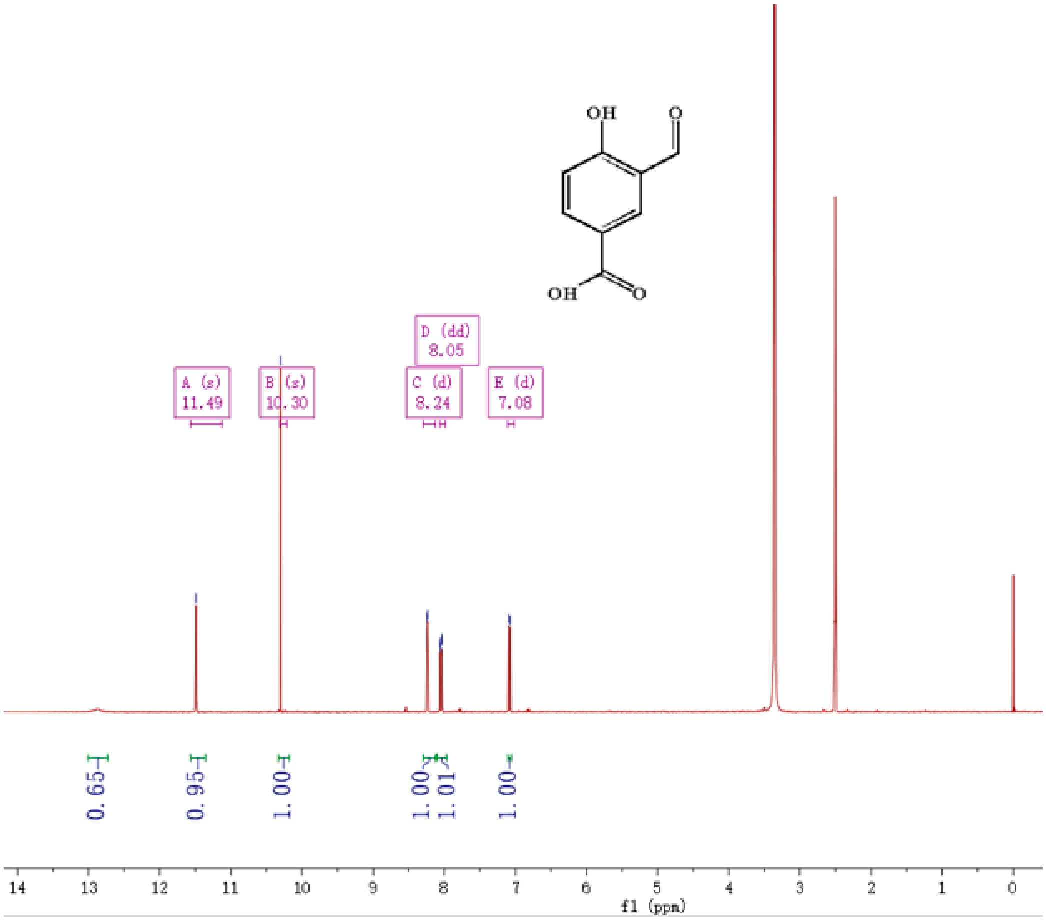

Figure S21 ^{1}H NMR of 3-formyl-4-hydroxybenzoic acid.
